# Distribution and Genetic Variability of *Bemisia tabaci* Cryptic Species (Hemiptera: Aleyrodidae) in Italy

**DOI:** 10.3390/insects12060521

**Published:** 2021-06-04

**Authors:** Sabrina Bertin, Giuseppe Parrella, Mauro Nannini, Giorgia Guercio, Elisa Troiano, Laura Tomassoli

**Affiliations:** 1 CREA Research Centre for Plant Protection and Certification, via C.G. Bertero 22, 00156 Rome, Italy; guerciogiorgia@gmail.com (G.G.); laura.tomassoli@crea.gov.it (L.T.); 2 National Research Council, Institute for Sustainable Plant Protection (CNR-IPSP), Piazzale Enrico Fermi 1, Napoli, 80055 Portici, Italy; giuseppe.parrella@ipsp.cnr.it (G.P.); elisa.troiano@ipsp.cnr.it (E.T.); 3 Agris Sardegna, Servizio Ricerca Studi Ambientali, Difesa delle Colture e Qualità Delle Produzioni, Viale Trieste 111, 09123 Cagliari, Italy; mnannini@agrisricerca.it

**Keywords:** Mediterranean (MED) species, Middle East–Asia Minor 1 (MEAM1) species, MEDQ haplogroups, begomoviruses, horticultural crops, ornamental crops

## Abstract

**Simple Summary:**

*Bemisia tabaci* is a key pest of horticultural, fibre and ornamental crops, primarily as a vector of plant viruses. This whitefly is considered as a complex of morphologically indistinguishable species that differ in several biological traits. In Italy, the Mediterranean (MED) and Middle East–Asia Minor 1 (MEAM1) species are known to be present in southern regions as well as in Sicily and Sardinia, where they have been responsible for economically important yield losses to horticultural crops since the 1990s. Recent reports of infestations in some areas of central Italy prompted a reassessment of the species distribution in the country, and several sites have been inspected at different central and southern regions and in the main islands. The survey confirmed that *B. tabaci* is nowadays established in central Italy even at more northern latitudes than those noticed before, with MED that is clearly prevailing on MEAM1 species throughout the country. The extensive presence of *B. tabaci* in Italy causes new concerns for the growing number of horticultural and ornamental productive sites that are potentially endangered by whitefly-transmitted viruses. The prevalence of the MED species is even more worrying for its ability to rapidly develop insecticide resistances.

**Abstract:**

*Bemisia tabaci* is a key pest of horticultural, fibre and ornamental crops worldwide, primarily as a vector of plant viruses. In Italy, *B. tabaci* has established since the 1980s–1990s in southern regions as well as in Sicily and Sardinia. Recent reports of infestations in some areas of central Italy prompted a new survey to assess the whitefly distribution in the country as well as to update the species and haplotype composition of the populations present in southern Italy and in the main islands. The survey confirmed that *B. tabaci* is nowadays established in central Italy even at more northern latitudes than those noticed before. Most of the specimens collected throughout the country belonged to the Mediterranean (MED) species. The MEDQ1 and Q2 haplogroups were prevailing in open-field and greenhouse cultivations, respectively, except in Sardinia where only Q1 specimens were found on a wide range of crops and weeds. Population genetics analyses showed that several MEDQ1 haplotypes currently occur in Italy and their distribution is unrelated to evident temporal and geographic trends, except for a new genetic variant which seems to have originated in Sardinia. The MED species is known to better adapt to insecticide treatments and high temperatures, and its northward spread in Italy may have been favoured by the intensive agricultural practices and steady increase in both winter and summer temperatures occurring in the last few decades. The extensive presence of *B. tabaci* in Italy proves that a strict surveillance for possible new outbreaks of whitefly-transmitted viruses should be addressed to a range of sites that are expanding northwards.

## 1. Introduction

*Bemisia tabaci* (Gennadius) (Hemiptera: Aleyrodidae) is a global pest that is responsible for economically important losses to horticultural, fibre and ornamental crops worldwide. Both direct and indirect damages are ascribable to the sap-sucking behaviour of *B. tabaci*, such as a reduction in the plant vigour, the abundant excretion of honeydew that favours the development of sooty moulds, and the transmission of hundreds of plant viruses mostly belonging to the genus *Begomovirus* (family Geminiviridae) [1,2].

Nowadays, *B. tabaci* is considered a complex of at least 40 morphologically indistinguishable species [3,4,5,6,7,8,9]. These cryptic species have been discriminated based on the mitochondrial *cytochrome oxidase subunit 1* (COI) sequence variability [3,10,11], but also differ in several biological traits such as the host–plant range [12,13,14], the induction of physiological plant disorders [15,16], insecticide resistance [17,18], invasiveness [19,20] and virus transmission efficiency [2,21], with most of these features influenced by the secondary endosymbionts harboured [22]. Most of the cryptic species are affiliated to continents, except the Mediterranean (MED) and Middle East–Asia Minor 1 (MEAM1), formerly referred to as biotype Q and B, respectively, that have spread all over the world in recent decades [23]. Although they have been found to co-exist in many areas, MED and MEAM1 evolved different adaptive traits and host preference through time [24,25]. For instance, MED populations took advantage of tolerance to high temperatures and the ability to rapidly develop insecticide resistances [14,17,18,26], whereas MEAM1 has been more competitive than MED in insecticide-free areas, thanks to reproductive interference mechanisms that increase its mating efficiency [27,28,29]. These different biological features led MED to become the predominant cryptic species where intensive farming is applied, and a progressive increase in MED infestations as well as a displacement of MEAM1 have been observed in several countries [30,31,32,33,34].

The successful adaptation of MEAM1 and MED to environmental and farming conditions has likely been supported by their genetic plasticity. Indeed, high genetic variability has been found at the intra-specific level and led to the definition of several COI haplotypes [35]. Within the MED species, more than eighty unique haplotypes have been noticed worldwide, and these have been split into four major groups ascribable to different home ranges and geographical distributions [35]. The Q1 and Q2 groups were originally present in western and eastern Mediterranean countries, respectively, and have recently spread to the rest of Europe, to the USA, and to Asia and South America [31,34,36,37,38,39,40,41]. The Q3 and African silver-leafing (ASL) groups include populations from the African home-range, being ASL from several sub-Saharan countries and Q3 from Burkina Faso only [42,43]. The MED groups have also showed differences in environmental and host adaptation, symbiont community and insecticide resistance [42,44,45], and the recent finding that the ASL group is reproductively isolated from Q1 and Q2 suggests a reassessment of the status of MED as a single species [46,47].

In Italy, *B. tabaci* became an important agricultural pest since the 1990s mainly in association with disease outbreaks caused by two whitefly-transmitted begomoviruses, tomato yellow leaf curl virus (TYLCV) and tomato yellow leaf curl Sardinia virus (TYLCSV) [48,49]. Both MED and MEAM1 cryptic species have been recorded in the southern regions of Italy, together with the minor species Italy (formerly referred to as biotype T) and its genetic variant Ru, which are present in uncultivated areas [32,50,51]. MED populations have progressively prevailed on MEAM1 in both greenhouses and open-field cultivations as well as on weeds [30,32,52,53]. Moreover, an increase in the frequency of the MED haplogroup Q2 (MEDQ2) has been reported in the last decade in the Campania region [54]. Along with the records from Spain and France [55], this confirms that MEDQ2 has shifted from the eastern (Syria, Israel, Turkey, Cyprus and Greece) to the western Mediterranean basin. A combination of climatic conditions and intensive agricultural practices has likely favoured the spread and the establishment in southern Italy of MEDQ2 populations that are known to be the most adapted to the high temperatures that can be reached during the summer, especially under plastic greenhouses [54].

The presence of *B. tabaci* in Italy has been for a long time restricted to the southern regions and the main islands Sicily and Sardinia. A recent study provided evidence of the establishment of whitefly populations also in central Italy, where the presence of *B. tabaci* was thought to be only occasional. Single and mixed populations of MED and MEAM1 were found in different horticultural areas of the Latium region, suggesting that the steady increase in the temperature which has occurred in the Mediterranean basin in the last decade may have favoured a northward spread of the species [56]. This finding raised new concerns for the possible presence of *B. tabaci* in the nearby regions of central Italy that have similar climatic and cultural conditions as well as for the introduction in free areas of whitefly-transmitted viruses, specially the emergent begomovirus tomato leaf curl New Delhi virus (ToLCNDV). This study presents the results of a recent survey carried out in different regions of central Italy to assess the current distribution of *B. tabaci* in the country. The study also includes further samplings in southern Italy as well as in Sicily and Sardinia to update the species and haplotype composition of the whitefly populations in Italy.

## 2. Materials and Methods

### 2.1. Sample Collection

**Figure 1:**
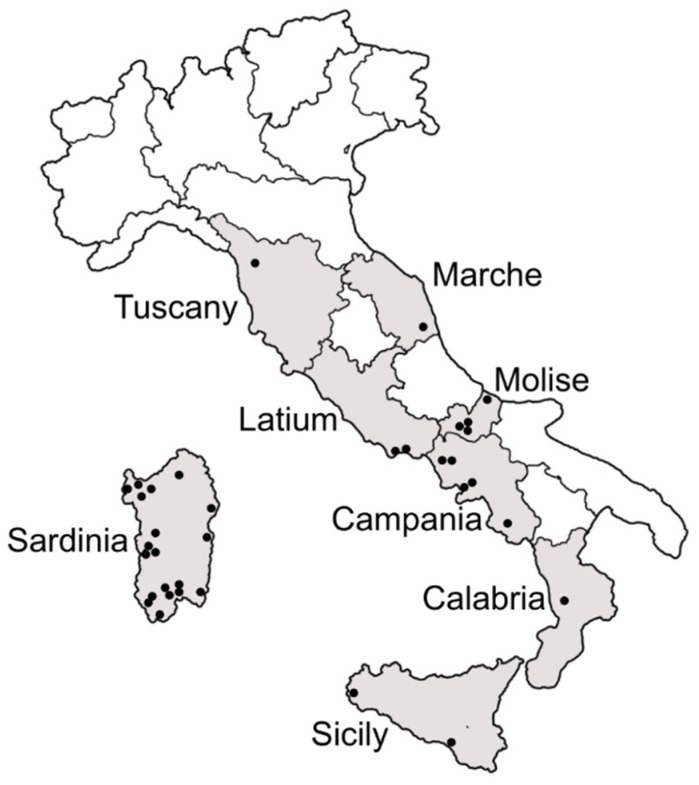
Map of Italy showing the regions and the localities (black dots) inspected for the presence of *Bemisia tabaci*.

Samples of *B. tabaci* were collected at different localities in Tuscany, Marche, Latium and Molise regions in central Italy, in Campania and Calabria regions in southern Italy and in the main islands Sicily and Sardinia (Figure 1). The surveys were carried out between 2017 and 2019, during the period from March to October. Samplings were performed on horticultural and ornamental crops cultivated in both open-field and protected conditions as well as on weeds. Sampling information is reported in Table S1. Whitefly adults were collected by means of a mouth aspirator and stored in 99% ethanol at 4 °C until the laboratory tests. The collected specimens were first examined through a stereo-microscope to exclude any contamination with other whitefly species, namely *Trialeurodes vaporariorum* (Westwood).

Each collection sample consisted of 5–50 individuals of *B. tabaci* collected at different sites on different host plants and at a different time. The DNA of two to ten *B. tabaci* specimens per collection sample was extracted and tested for the identification of the *B. tabaci* species and haplotypes.

### 2.2. Molecular Analyses for the Identification of B. tabaci Species and Haplogroups

The DNA was extracted from single individuals of *B. tabaci* using the Xpert directXtract Lysis Buffer (GRiSP Research Solutions, Porto, Portugal). Each whitefly was ground in 50 µL of reaction mixture (35 μL PCR-grade ddH_2_O, 10 μL Xpert directXtract buffer A, and 5 μL Xpert directXtract buffer B) and the DNA was extracted following the manufacturer’s instructions.

The identification of *B. tabaci* cryptic species as well as the MED Q1 and Q2 haplogroups was carried out by means of the PCR-RFLP assay suggested by [32]. The discriminating 866-bp COI region was amplified using the primers C1-J-2195 and TL2-N-3014 [57]. The PCR was performed in 25 µL reaction volume containing: 2 X PCR Master Mix (Promega, Madison, WI, USA), 2 mM MgCl_2_, 0.5 µM of each primer and 1 µL of DNA. The thermo-cycling conditions consisted of an initial denaturation cycle at 94 °C for 5 min, 35 cycles at 95 °C for 30 s, 45 °C for 45 s and 72 °C for 1 min and a final cycle at 72 °C for 10 min. The amplified DNA was analyzed by electrophoresis onto a 1% (*w*/*v*) agarose gel and then digested with the restriction enzyme ApoI according to the manufacturer’s instructions (New England BioLabs, Ipswich, MA, USA). The restriction fragments were separated by electrophoresis onto 2% (*w*/*v*) 1 × TBE agarose gels (1.5% MetaPhorTM agarose for resolution of small nucleic acids + 0.5% SeaKem^®^ LE Agarose; Lonza, Basel, Switzerland) at 70 V for 2 h and stained with ethidium bromide.

MEAM1 and MED specimens representative of each sampling area were selected, and their COI amplicons were purified through Amicon^®^ Ultra-0.5 Centrifuge Filter Devices (Merck KGaA, Darmstadt, Germany) and sequenced in both directions. The sequences were assembled using MEGA (version X) [58] and deposited in GenBank database (NCBI – NIH, Bethesda, MD, USA) with the accession numbers MW604141-MW604196 (Table S1).

### 2.3. Sequence Analyses

Phylogenetic analyses were conducted using MEGA. The COI sequences were aligned by MUSCLE together with sequences that have been chosen in GenBank to be representative of the worldwide MEAM1 and MED haplotypes. A maximum-likelihood (ML) consensus tree was generated from 1000 bootstrap replicates. The Tamura–Nei model with five gamma distribution categories (TN93 + G) was the best-fit model selected according to BIC and AIC criteria using Model Selection analysis as implemented by MEGA. The COI sequence of *Bemisia afer* was used as an outgroup (GenBank accession number: GU220055).

The COI sequences of MED specimens were analysed in the DnaSP package v. 6.12.03 [59] to estimate the number of haplotypes, the haplotype diversity (Hd) and the nucleotide diversity per site (π), and to perform the neutrality Fu’s (*Fs*) and Tajima’s (*D*) tests. The geographic structure of the MEDQ1 haplotypes was assessed by Median Joining networks (MJN) [60] that were constructed using the PopART software [61]. The resulting networks identify the relationship between the different haplotypes as well as the significant number of substitutions connecting haplotypes. MEDQ1 samples from Sicily were excluded since they were underrepresented (Table S1). The same network analysis was performed to infer a possible ancestor–descendant relationship among the MEDQ1 haplotypes. In this case, available COI sequences of MEDQ1 specimens previously collected in Latium (GenBank accession numbers: MF447849, MW380736 and MW380737) [57] and Campania (FN557444-FN557455, FN557457, FN557458, FN557464, FN557465, FN557468, FN557469) [32] were included.

## 3. Results

### 3.1. Presence and Distribution of B. tabaci Species Complex

Samples of *B. tabaci* were collected in all the inspected localities in central and southern Italy as well as in the main islands (Figure 1; Table S1). The partial mtCOI gene fragments were obtained from a total of 403 *B. tabaci* specimens: 34 from Tuscany, 23 from Marche, 48 from Latium, 36 from Molise, 15 from Campania, 6 from Calabria, 221 from Sardinia and 20 from Sicily. The PCR-RFLP profiles showed that most of the individuals belonged to the Q1 and Q2 mitochondrial groups within the MED species; only four specimens belonged to the MEAM1 species, one from Molise and three from Sardinia (Table S1).

In the Tuscany region, MEDQ2 populations and MEDQ1-Q2 mixed populations were found in several greenhouses in Pescia on solanaceous, cucurbit and ornamental crops. A cucurbit field (zucchini and pumpkin crops) was also inspected, providing evidence of the presence of *T. vaporariorum* only. In the Marche region, several host plants belonging to Asteraceae, Solanaceae and Brassicaceae families were surveyed in open fields, and the MEDQ1 group was abundantly found alone or together with MEDQ2. In the Latium region, MEDQ1-Q2 mixed populations were frequently sampled at different sites in the Agro Pontino horticultural area on cucurbit crops cultivated in both protected and open-field conditions. The MEDQ2 group was more abundant than MEDQ1 and was the only MED haplotype collected on pepper. A prevalence of the MEDQ2 group was also observed during the surveys in the Molise region. Here, MEDQ2 specimens were collected on ornamental crops cultivated in protected conditions at different localities and were found together with MEDQ1 in a *Solanum melongena* field cultivated in Petacciano, where a tomato field as well as two open gardens hosting vases of Laurus spp. and Viburnum spp. were also inspected but only *T. vaporariorum* was sampled. In the Campania and Calabria regions, southern Italy, single and mixed populations of both MEDQ1 and MEDQ2 groups were collected on cucurbits cultivated in open fields as well as on weeds. In Sardinia, apart from a few specimens belonging to MEAM1 species, only the MEDQ1 variant was found at several localities throughout the region on a large variety of weeds and crops in both greenhouses and open fields. Instead, MEDQ2 was almost the only mitochondrial type collected in the greenhouses inspected in Marsala and Gela, two distant areas in Sicily.

### 3.2. Genetic Variability within the MED Species

**Figure 2:**
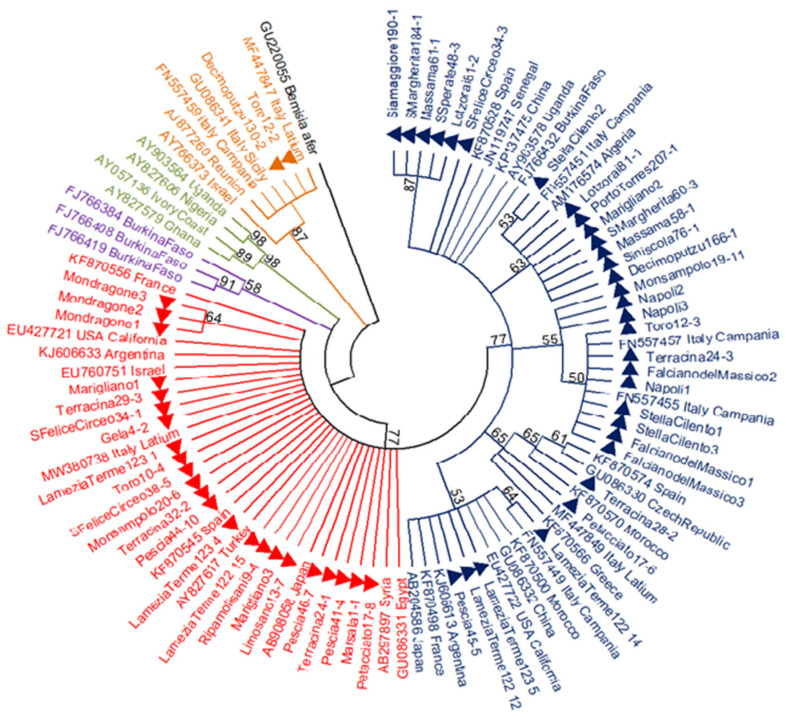
Maximum-likelihood (ML) majority rule tree obtained using 866-bp mtCOI sequences of 56 *Bemisia tabaci* specimens collected in this study (filled triangles) and those sourced from GenBank, for a total of 92 sequences. A COI sequence of *Bemisia afer* was used as the outgroup. The consensus tree was obtained after 1000 bootstrap replicates; branches with <50% bootstrap values are collapsed. Colours represent the different species and haplogroups of *B. tabaci*: MEAM1, orange; MEDQ1, dark blue; MEDQ2, red; MEDQ3, purple; MEDASL, green.

The 866-bp mtCOI sequences obtained from two MEAM1 and 54 MED individuals collected in the different sampling areas were analysed in a phylogenetic tree including several *B. tabaci* sequences worldwide (Figure 2). Within the MED species, the sequences from the Q2 group were poorly divergent and they gave origin to one clade of monophyletic branches that frequently collapsed into polytomies in the majority rule tree, except for the group of specimens from Mondragone (Campania region). Only three different haplotypes were found among the 23 MEDQ2 samples from Italy and they showed low haplotype and nucleotide diversity values (Hd = 0.312 ± 0.115 SD; π = 0.0004 ± 0.0001 SD).

**Figure 3:**
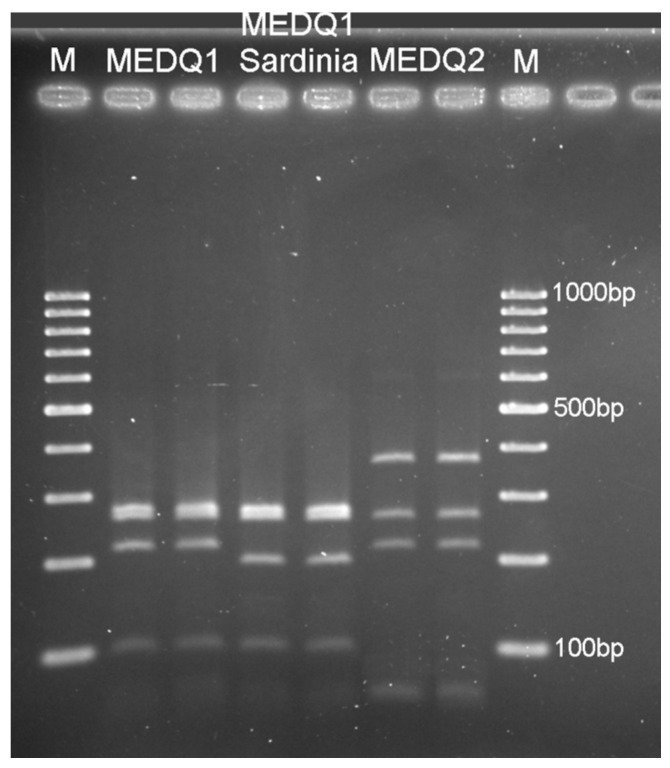
PCR-RFLP profile after digestion with ApoI endonuclease of the COI-PCR product for the following MED haplotypes of *Bemisia tabaci*: MEDQ1 (expected fragment sizes: ~44, 92, 212, 252, 266 bp, corresponding to the cutting sites at the positions 252, 296, 562 and 654), Sardinian MEDQ1 (~18 not visible, 44, 92, 194, 252, 266 bp, corresponding to the cutting sites at the positions 252, 296, 562, 654 and 672) and MEDQ2 (~44, 212, 252, 358 bp, corresponding to the cutting sites at the positions 252, 296 and 654); M: molecular weight marker (100 bp ladder).

The MEDQ1 sequences showed higher levels of genetic variability and were distributed into different clusters (Figure 2). The cluster supported by the highest bootstrap value (87%) was composed of five samples from Sardinia only: two samples collected in south (Santa Margherita 184-1, accession number MW604184; San Sperate 48-3, MW604186), two in central-west (Siamaggiore 190-1, MW604183; Massama 61-1, MW604185) and one in central-east (Lotzorai 81-2, MW604182) Sardinia. These COI sequences were also characterised by one more ApoI cutting site at position 672 bp and had a PCR-RFLP pattern that is slightly different from the other known MEDQ1 haplotypes (Figure 3).

**Figure 4:**
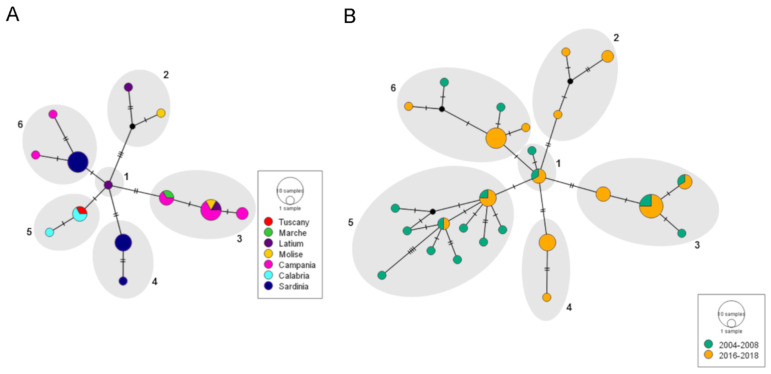
Median Joining haplotype networks showing (**A**) the geographic relationship among thirteen MEDQ1 haplotypes of *Bemisia tabaci* collected in Italy between 2017 and 2018; (**B**) the temporal relationship among the MEDQ1 haplotypes of *B. tabaci* at two collection periods, 2004–2008 and 2016–2018. The main haplotype groups (1–6) are hand-drawn to assist in visualisation.

Among the 31 MEDQ1 COI sequences obtained in this study, a total of 13 haplotypes were identified. The haplotypes showed a high diversity value (Hd = 0.908 ± 0.027 SD) that did not correspond to such high levels of nucleotide diversity per site (π = 0.004 ± 0.0004 SD), suggesting that this genetic variability is poorly fixed in the MEDQ1 populations. The neutrality test did not provide evidence of any demographic trends, since both Fu’s and Tajima’s tests had negative but not significant values (Fs: −3.114, *p* > 0.10; D: −0.84315, *p* > 0.10). A possible structure of the MEDQ1 populations was inferred by grouping the sequences by region of origin in a haplotype network analysis. The 13 MEDQ1 haplotypes were arranged in a star-like network made of six main groups (Figure 4A). Most of these groups included haplotypes from different regions, indicating that MEDQ1 has a small geographical structure. Only group 4, which is composed of the genetic variant from Sardinia described above, showed a correlation between genetic variability and region of origin. The other samples from Sardinia all gathered in group 6 together with two haplotypes from Campania. Group 3 is the most represented within the network and includes samples from Campania, Molise, Latium and Marche, indicating that these haplotypes are widespread in Italy.

A second network analysis, including sequences from samples previously collected in the Latium and Campania regions, was carried out to investigate a possible temporal evolution of MEDQ1 in Italy. A total of 52 sequences were analysed and divided into two categories: one including 18 samples collected in Campania between 2004 and 2008 and one including 34 samples collected between 2016 and 2018 in all the other regions (31 from this study and 3 collected in Latium in 2016). The result is still a star-like network with no evident evolutive genetic trends. The network is made of six main haplotype groups that overlapped the geographical groups (Figure 4B) and shows that most of the haplotypes recorded in 2016–2018 were already present in Campania in 2004–2008. Taken together, the demography and network analyses provided evidence that the Italian MEDQ1 haplotypes are neutrally evolving, and neither selection pressures leading to a population structure nor recent demographic expansions can be inferred.

## 4. Discussion

In agriculture, species invasions are among the drivers for changes in natural equilibria and have often been a major threat for crop production and management. The principal characteristic of an invading species is its ability to adapt rapidly to environmental changes, and such adaptation has often genetic bases. *Bemisia tabaci* has become a paradigm for both invasiveness and genetic variability in several countries worldwide, and in Italy as well. After the first introductions in the 1980s–1990s [48,62], populations of *B. tabaci* rapidly established at a high density level in Sicily, Sardinia and southern Italy and then progressively moved northwards. The spread in new areas occurred together with a genetic change of *B. tabaci* populations that gradually shifted from MEAM1 to MED cryptic species. This study provides evidence that the whitefly is nowadays established in central Italy and that the MEAM1 species occurs at very low levels, confirming that in Italy the geographic and genetic status of *B. tabaci* are still changing.

The main aim of the study was to extend the search for *B. tabaci* in central Italy, after its first occurrence in Latium in 2016 [56]. The new surveys showed the establishment of whitefly populations at more northern latitudes than those previously inspected, such as in the Tuscany and Marche regions. Almost all the specimens collected in the central regions belonged to the MED species, whereas in 2016–7 a consistent population of MEAM1 species was found in a location in northern Latium (Viterbo district). In that case, the less intensive agricultural practices and the prevalence of open-field cultivations were thought to create an environmental niche still favourable to MEAM1 and where the MED invasion has not occurred yet [56]. Similar agricultural conditions were also found during this survey in other areas of central Italy, such as Mosampolo del Tronto (Marche region) and Petacciano (Molise region), but in these cases only MED specimens were collected. This suggests that in central Italy the MED species is increasingly adapting even to the outdoor conditions and that its spread from protected to field cultivations is more frequent than it was previously thought [48,56]. The adaptative process could have been favoured not only by the high genetic plasticity of the MED species but also by the increase in both winter and summer temperatures which recently occurred in the Mediterranean area. Indeed, several prediction models simulating the potential distribution of the *B. tabaci* species under temperature changes have proved that global climate warming would expand the northern boundaries of the open-field populations in Europe [63,64].

Both MEDQ1 and MEDQ2 mitochondrial variants were found in single or mixed populations in central and southern continental Italy. Most of the specimens collected in greenhouse crops belonged to MEDQ2 (87.6%), whereas MEDQ1 was prevailing in open-field cultivations and weeds (60%). These rates confirmed the previous observations conducted in southern Italy as well as in other Mediterranean countries that MEDQ2 is most adapted to protected conditions [31,54]. Indeed, the MEDQ2 populations are likely to be more tolerant to the high temperatures that can be reached during the summer under the greenhouses and to the insecticides that are widely applied on vegetable and ornamental protected crops. The surveys carried out in Sicily confirmed the high incidence of MEDQ2 in protected conditions.

A different scenario was observed in Sardinia, where only the MEDQ1 mitochondrial group was found. This suggests that the MED specimens previously found in Sardinia since the late 1990s together with MEAM1 [32,50] belonged to the Q1 haplogroup and they later spread throughout the island, replacing the co-present species. The factors that have driven the establishment of MEDQ1 rather than MEDQ2 populations are not completely evident. The temperature trends of the island are comparable with those recorded in southern Italy and thus compatible with the MEDQ2 settlement. Moreover, the samplings have included several sites with environmental and agricultural conditions that are apparently favourable to MEDQ2, such as the important horticultural district in southern Sardinia (Decimoputzu and surrounding localities) where the high density of greenhouses and intensive crop management could potentially select MEDQ2 populations [54,65]. Additionally, the Sardinian samples were from a wide range of crops, including *S. melongena*, *Solanum nigrum* and *Brassica oleracea*, that have been suggested as preferred hosts of MEDQ2 [45,54]. We can therefore hypothesise that MEDQ2 has not likely been introduced into the island yet. This could be related to the fact that Sardinia is geographically distant from the continent and is less exposed to constant flows of plant trades in favour of local nurseries compared to Sicily, which is one of the most important access points of horticultural material in the Mediterranean basin. This may have reduced the chances of MEDQ2 introduction and allowed the MEDQ1 populations to become durably widespread throughout the island.

Among the four mitochondrial variants that have been identified within MED cryptic species so far, Q1 shows the highest level of genetic variability and includes several haplotypes worldwide [24,33,35,39,66]. A population genetics study carried out on whitefly populations from several Mediterranean countries (France, Spain, Morocco, Tunisia and Greece) identified at least 21 MED haplotypes. Among these, 18 were from the MEDQ1 group and only three from MEDQ2. The present study confirms that MEDQ1 also accounts for the highest level of genetic variability within the MED populations of Italy, being most of the MEDQ2 specimens belonging to only one haplotype. The different degree of genetic differentiation between the two mitochondrial groups can likely be related to the fact that MEDQ1 was originally present in the western Mediterranean area, whereas MEDQ2 settled in the same area more recently [55,56,66]. The 13 MEDQ1 haplotypes identified in Italy have little nucleotide variability and it seems that they did not undergo any evident evolutive trends, neither selection nor expansion. Indeed, most of the haplotypes found between 2016 and 2018 were shared with whiteflies collected ten to fourteen years before, suggesting that no selection has occurred during that time. Moreover, the genetic diversity was not correlated with the regions of origin, except for the samples from Sardinia. The MEDQ1 specimens collected on the island were represented by two haplotype groups, one that shares similar sequences with only few specimens from Campania and one that constitutes a genetic variant that was not noticed before. We can hypothesise that in Sardinia the long-lasting presence of the MEDQ1 type only may have reduced the interbreeding processes and suppressed the genetic variability.

In general, the absence of both temporal and geographic genetic structures suggests that the MEDQ1 haplotypes in Italy have neutrally evolved in the last few years, and they have likely originated from multiple introductions independently occurring at different sites and times rather than from selection events. The absence of selection also implies that all the MEDQ1 haplotypes are potentially able to adapt to different conditions and would establish even further north as soon as the climatic conditions will become favourable. The invasiveness of a particular genotype of *B. tabaci* might also depend on the phenotype of inherited bacterial endosymbionts, which are known to enhance insecticide susceptibility [67], facilitate virus transmission [68] or confer tolerance to high temperatures [69]. The endosymbiont composition has been found to differ in some MEDQ1 and MEDQ2 populations in southern Italy, and a possible role in MEDQ2 spread in this area has already been postulated [54]. The further characterisation of the secondary endosymbionts associated with the different MED haplotypes found in several areas of Italy would help in better predicting the spreading potential of *B. tabaci* in this country.

This study defines a new map of the *B. tabaci* populations in Italy and states that this pest is now established at latitudes where it was previously unnoticed or thought to be only occasional. This extensive presence causes new concerns for a wider number of horticultural and ornamental productive sites that are potentially endangered by *B. tabaci*-transmitted viruses. The fact that MED is prevailing over MEAM1 species should alarm even more, not only for its higher insecticide resistance but also because it has been suggested that MED can be more efficient than MEAM1 in begomovirus transmission [70,71]. Currently, the major threat is represented by the diffusion of ToLCNDV, an emergent begomovirus that has been categorised in 2019 as a quarantine pest (EU Regulation 2019/2072), being very harmful mainly to cucurbit crops [72,73]. Thus far, outbreaks of ToLCNDV have been reported in Sicily, Sardinia and in several regions of southern Italy, where the previous presence of the begomoviruses TYLCV and TYLCSV can increase risks that new genetic recombinants or new viral species will arise over time, especially in the common weed hosts [74,75]. In this survey, begomovirus infections actually occurred at the time of samplings in those areas where *B. tabaci* populations have been established over the years [32,50,56]. For instance, the cucurbit cultivations surveyed in Latium, Calabria, Campania (at Mondragone and Stella Cilento localities) and Sardinia were infected by ToLCNDV, and the TYLCV/TYLCSV complex was widespread in the tomato cultivations inspected in Sardinia. The presence of *B. tabaci* in regions of central Italy makes the introduction of begomoviruses also possible in further horticultural areas with important economic consequences. This has already occurred within the Latium region, where the ToLCNDV outbreaks, initially restricted to the Agro Pontino area, also occurred in the northern area of Agro Romano [76] where *B. tabaci* populations had recently been noticed [56]. This survey proves that in Italy the strict surveillance for possible new outbreaks of *B. tabaci*-transmitted viruses should be addressed to a range of sites that are expanding northwards. In the same range, all the integrated management strategies should be adopted to keep *B. tabaci* under control even where the whitefly is not regarded as a harmful pest yet.

## Supplementary Materials

The following is available online at https://www.mdpi.com/article/10.3390/insects12060521/s1, Table S1: Samples of *Bemisia tabaci* collected in several Italian regions between March 2017 and September 2019 and their genetic characterisation at species and haplogroup level. The GenBank accession numbers refer to the COI sequences of MEAM1, MEDQ1 and MEDQ2 specimens that are representative of each collection sample.
