# Supplementary material for: Distribution and Genetic Variability of Bemisia tabaci Cryptic Species (Hemiptera: Aleyrodidae) in Italy
Source: Insects. 2021 Jun 4;12(6):521. doi: 10.3390/insects12060521 (PMC8229085; doi:10.3390/insects12060521)
Supplement: Supplementary file 1 [file insects-12-00521-s001.zip › insects-1242141-SI.pdf]

Table S1. Samples of *Bemisia tabaci* collected in several Italian regions between March 2017 and September 2019 and their genetic characterization at species and haplogroup level. The GenBank accession numbers refer to the COI sequences of MEAM1, MEDQ1 and MEDQ2 specimens that are representative of each collection sample.

Italian region	Sampling site[1]	Date of sampling	Host plant	Type of cultivation	Species and haplogroup[2]	GenBank accession number
**Tuscany** (central Italy)	Pescia 43°54'N 10°41'E	October 2018	*Capsicum annuum* L.	Greenhouse	MEDQ2 (4)	MW604141
			Cucurbita moschata Duch.	Greenhouse	MEDQ2 (5)	
			Cannabis sativa L.	Greenhouse	MEDQ2 (5)	
	Pescia 43°51'N 10°41'E	October 2018	*Capsicum annuum* L.	Greenhouse	MEDQ1 (1)	
					MEDQ2 (8)	MW604143
	Pescia 43°53'N 10°41'E	October 2018	*Mandevilla* spp.	Greenhouse	MEDQ1 (2)	MW604142
					MEDQ2 (3)	
			Euphorbia pulcherrima Will.	Greenhouse	MEDQ1 (2)	
					MEDQ2 (4)	MW604144
**Marche** (central Italy)	Monsampolo del Tronto 42°88'N 13°80'E	September 2018	*Helianthus tuberosus* L.	Open field	MEDQ1 (11)	MW604146
			Solanum melongena L.	Open field	MEDQ1 (5)	
					MEDQ2 (5)	MW604145
			Brassica oleracea L.	Open field	MEDQ1 (2)	
**Latium** (central Italy)	Terracina 41°32'N 13°11'E	October 2018	*Cucurbita pepo* L.	Greenhouse	MEDQ1 (1)	MW604148
					MEDQ2 (6)	MW604152
	Terracina 41°29'N 13°09'E	October 2018	*Cucurbita pepo* L.	Greenhouse	MEDQ1 (1)	MW604149
					MEDQ2 (10)	MW604153
			Capsicum annuum L.	Greenhouse	MEDQ2 (9)	MW604154
		October 2018	*Cucurbita pepo* L.	Open field	MEDQ1 (2)	MW604147

	San Felice Circeo<br>41°28’N 13°09’E				MEDQ2 (9)	MW604150
	San Felice Circeo<br>41°28’N 13°08’E	October 2018	*Cucurbita pepo* L.	Greenhouse	MEDQ1 (2)<br>MEDQ2 (8)	 MW604151
**Molise**<br>(southern Italy)	Ripalimolisani<br>41°64’N 14°64’E	September 2018	*Mandevilla* spp.	Greenhouse	MEDQ2 (7)	MW604159
	Toro<br>41°56N 14°78’E	September 2018	*Mandevilla* spp.<br>*Aralia* spp.<br>*Capsicum annuum* L.	Greenhouse<br>Greenhouse<br>Greenhouse	MEDQ2 (5)<br>MEDQ2 (2)<br>MEAM1 (1)<br>MEDQ1 (2)<br>MEDQ2 (1)	MW604160<br><br>MW604196<br>MW604157
	Limosano<br>41°67’N 14°65’E	September 2018	*Begonia* spp.	Greenhouse	MEDQ2 (8)	MW604156
	Petacciato<br>42°03’N 14°85’E	September 2018	*Solanum melongena* L.	Open field	MEDQ1 (6)<br>MEDQ2 (4)	MW604158<br>MW604155
**Campania**<br>(southern Italy)	Falciano del Massico<br>41°08’N 13°56’E	August 2018	*Cucumis melo* L.	Open field	MEDQ1 (3)	MW604173-5
	Mondragone<br>41°06’N 13°40’E	September 2019	*Cucurbita pepo* L.	Open field	MEDQ2 (3)	MW604161-3
	Stella Cilento<br>40°22’N 15°09E	August 2019	*Cucurbita moschata*<br>Duchesne ex Poir.	Open field	MEDQ1 (3)	MW604167-8<br>MW604170
	Napoli<br>40°50’N 14°13’	October 2018	*Ipomoea indica* (Burm.f.)<br>Merr.	Wild	MEDQ1 (3)	MW604169<br>MW604171-2
	Marigliano<br>40°57’N 14°26’E	September 2018	*Ipomoea* spp.	Wild	MEDQ1 (1)<br>MEDQ2 (2)	MW604165<br>MW604164<br>MW604166
**Calabria**		October 2017	*Cucurbita pepo* L.	Open field	MEDQ1 (2)	MW604180-1

(southern Italy)	Lamezia Terme 38°82’N 16°26’E				MEDQ2 (1)	MW604178
	Lamezia Terme 38°83’N 16°23’E	October 2017	*Cucurbita pepo* L.	Open field	MEDQ1 (1)	MW604179
					MEDQ2 (2)	MW604176-7
**Sardinia** (island) Southern Sardinia: Sud Sardegna and Cagliari provinces	Decimoputzu 39°33’N 8°90’E	March 2017	*Cucurbita pepo* L.	Greenhouse	MEDQ1 (7)	
		May 2017	*Conyza* spp., *Solanum nigrum* L., *Malva sylvestris* L.	Open field	MEDQ1 (3)	
		July 2018	*Solanum melongena* L.	Open field	MEDQ1 (5)	
			Solanum lycopersicum L.	Open field	MEDQ1 (3)	
	Decimoputzu 39°35’N 8°88’E	June 2017	*Capsicum annuum* L.	Greenhouse	MEDQ1 (2)	
		August 2017	*Sonchus* spp.	Open field	MEDQ1 (3)	
			Cucurbita moschata Duch	Open field	MEDQ1 (5)	
			Cucumis melo L.	Open field	MEDQ1 (4)	
		September 2017	*Solanum lycopersicum* L.	Greenhouse	MEDQ1 (3)	MW604192
	Decimoputzu 39°36’N 8°90’E	June 2017	*Cucurbita pepo* L.	Greenhouse	MEDQ1 (3)	
		August 2017	*Cucumis sativus* L.	Greenhouse	MEAM1 (1)	MW604195
					MEDQ1 (4)	
		September 2017	*Cucumis sativus* L.	Greenhouse	MEAM1 (1)	
					MEDQ1 (4)	
			Cucurbita pepo L.	Open field	MEDQ1 (5)	
	Decimoputzu 39°34’N 8°92’E	July 2018	*Cucumis melo* L.	Open field	MEDQ1 (2)	
			Citrullus lanatus L.	Open field	MEDQ1 (3)	
	San Sperate 39°36’N 9°00’E	May 2017	*Lantana* spp.	Open field	MEDQ1 (3)	MW604186
	San Giovanni Suergiu	September 2018	*Solanum melongena* L.	Open field	MEDQ1 (3)	

	39°06’N 8°56’E					
	Giba 39°06’N 8°63’E	September 2018	*Cucurbita pepo* L.	Open field	MEDQ1 (3)	
	Ussana 39°40’N 9°08’E	September 2018	*Solanum melongena* L.	Open field	MEDQ1 (3)	
	Donori 39°45’N 9°10’E	October 2018	*Cucurbita pepo* L.	Open field	MEDQ1 (4)	
	S. Margherita di Pula 38°97’N 8°97’E	May 2017	*Cucumis sativus* L.	Greenhouse	MEDQ1 (2)	
			Solanum lycopersicum L.	Greenhouse	MEDQ1 (3)	
		July 2017	*Chenopodium* spp.	Greenhouse	MEDQ1 (3)	
		August 2017	*Solanum nigrum* L.	Greenhouse	MEDQ1 (3)	
			Cucurbita pepo L.	Open field	MEDQ1 (2)	
			Capsicum annuum L.	Open field	MEDQ1 (3)	
			Amaranthus spp.	Open field	MEDQ1 (2)	
		September 2017	*Solanum melongena* L.	Open field	MEDQ1 (3)	
			Malva sylvestris L.	Open field	MEAM1 (1)	
					MEDQ1 (2)	MW604184
		August 2018	*Cucurbita pepo* L.	Greenhouse	MEDQ1 (7)	MW604187
			Capsicum annuum L.	Greenhouse	MEDQ1 (3)	
	S. Margherita di Pula 38°95’N 8°95’E	August 2017	*Cucumis melo* L.	Greenhouse	MEDQ1 (3)	
			Conyza spp.	Greenhouse	MEDQ1 (3)	
	Muravera 39°42’N 9°57’E	October 2018	*Solanum melongena* L.	Open field	MEDQ1 (3)	
**Sardinia** (island)	Massama 39°95’N 8°58’E	May 2017	*Capsicum annuum* L.	Open field	MEDQ1 (3)	
			Solanum melongena L.	Open field	MEDQ1 (3)	
			Cucurbita pepo L.	Open field	MEDQ1 (3)	MW604185

Central-Western Sardinia: Oristano province		July 2017	*Cucumis sativus* L.	Open field	MEDQ1 (3)	
		September 2017	*Cucumis melo* L.	Open field	MEDQ1 (3)	
		October 2017	*Solanum lycopersicum* L.	Open field	MEDQ1 (3)	
		August 2018	*Cucurbita pepo* L.	Open field	MEDQ1 (5)	MW604189
	Paulilatino 40°08'N 8°76'E	October 2018	*Cucurbita pepo* L.	Open field	MEDQ1 (3)	
	Nuraxinieddu 39°93'N 8°59'E	August 2018	*Solanum lycopersicum* L.	Open field	MEDQ1 (3)	
	Siamaggiore 39°93'N 8°63'E	May 2017	*Solanum lycopersicum* L.	Greenhouse	MEDQ1 (3)	
		August 2017	*Solanum lycopersicum* L.	Greenhouse	MEDQ1 (3)	
		October 2017	*Solanum lycopersicum* L.	Greenhouse	MEDQ1 (2)	
			Solanum melongena L..	Greenhouse	MEDQ1 (3)	
			Malva sylvestris L.	Greenhouse	MEDQ1 (3)	MW604183
**Sardinia** (island) Central-Eastern Sardinia: Nuoro province	Siniscola 40°59'N 9°72'E	September 2018	*Cucurbita pepo* L.	Open field	MEDQ1 (5)	MW604188
	Lotzorai 39°97'N 9°65'E	October 2018	*Cucumis sativus* L.	Open field	MEDQ1 (3)	MW604182 MW604190
**Sardinia** (island) Northern Sardinia: Sassari and Tempio Pausania provinces	Sorso 40°82'N 8°57'E	June 2017	*Solanum lycopersicum* L.	Greenhouse	MEDQ1 (3)	
		September 2017	*Phaseolus vulgaris L.*	Greenhouse	MEDQ1 (3)	
	Sorso 40°80'N 8°49'E	June 2017	*Cucurbita pepo* L.	Open field	MEDQ1 (3)	
			Solanum melongena L..	Open field	MEDQ1 (3)	
		July 2017	*Cucurbita pepo* L.	Open field	MEDQ1 (2)	
		September 2017	*Cucumis melo* L.	Open field	MEDQ1 (3)	
	Porto Torres 40°82'N 8°38'E	July 2017	*Solanum melongena* L..	Open field	MEDQ1 (3)	
			Cucumis melo L.	Open field	MEDQ1 (3)	
		September 2017	*Cucumis sativus* L.	Open field	MEDQ1 (3)	

		October 2017	*Cucurbita pepo* L.	Open field	MEDQ1 (3)	
			Capsicum annuum L.	Open field	MEDQ1 (3)	
			Solanum lycopersicum L.	Open field	MEDQ1 (3)	
		March 2018	*Brassica oleracea*	Open field	MEDQ1 (3)	MW604191
	Sassari (Agliadò)<br>40°80'N 8°49'E	August 2018	*Cucurbita pepo* L.	Open field	MEDQ1 (8)	
	S, Maria la Palma<br>40°66'N 8°25'E	September 2018	*Cucumis melo* L.	Open field	MEDQ1 (3)	
	Tempio Pausania<br>40°92'N 9°08'E	October 2018	*Solanum melongena* L.	Open field	MEDQ1 (4)	
**Sicily** (island)	Marsala<br>37°42'N 12°34'E	May 2019	*Capsicum annuum* L.	Greenhouse	MEDQ1 (1)<br>MEDQ2 (9)	 MW604193
	Gela<br>36°98'N 14°37'E	May 2019	*Capsicum annuum* L.	Greenhouse	MEDQ2 (10)	MW604194

[1] locality and geographical coordinates.
[2] Number of analysed specimens in brackets.
